# Optimising window size of semantic of classification model for identification of in-text citations based on context and intent

**DOI:** 10.1371/journal.pone.0309862

**Published:** 2025-03-24

**Authors:** Arshad Iqbal, Abdul Shahid, Muhammad Roman, Muhammad Tanvir Afzal, Umair ul Hassan

**Affiliations:** 1 Institute of Computing Kohat University of Science and Technology, Kohat, Pakistan; 2 National College of Ireland, Dublin, Ireland; 3 Shifa School of Computing, Shifa Tameer-e-Millat University, Islamabad, Pakistan; 4 JE Cairnes School of Business and Economics, University of Galway, Galway, Ireland; 5 Insight SFI Research Centre for Data Analytics, University of Galway, Galway, Ireland; Shifa Tameer-e-Millat University, PAKISTAN

## Abstract

Citations in scientific literature act as channels for the sharing, transfer, and development of scientific knowledge. However, not all citations hold the same significance. Numerous taxonomies and machine learning models have been developed to analyze citations, but they often overlook the internal context of these citations. Moreover, it is worth noting that selecting the appropriate word embedding and classification models is crucial for achieving superior results. Word embeddings offer n-dimensional distributed representations of text, striving to capture the nuanced meanings of words. Deep learning-based word embedding techniques have garnered significant attention and found application in various Natural Language Processing (NLP) tasks, including text classification, sentiment analysis, and citation analysis. Current state-of-the-art techniques often use small datasets with fixed window sizes, resulting in the loss of contextual meaning. This study leverages two benchmark datasets encompassing a substantial volume of in-text citations to guide the selection of an optimal word embedding window size and classification approaches. A comparative analysis of various window sizes for in-text citations is conducted to identify crucial citations effectively. Additionally, Word2Vec embedding is employed in conjunction with deep learning models and machine learning models such as Convolutional Neural Networks (CNNs), Gated Recurrent Units (GRUs), Long Short-Term Memory (LSTM) networks, Support Vector Machines (SVM), Decision Trees, and Naive Bayes.The evaluation employs precision, recall, F1-score, and accuracy metrics for each combination of window sizes. The findings reveal that, particularly for lengthy in-text citations, larger citation windows are more adept at capturing the semantic essence of the references. Within the scope of this study, window sizes of 10 achieve superior accuracy and precision with both machine and deep learning models.

## 1 Introduction

Scientific advancements are intrinsically tied to the cumulative body of research within a particular domain, and this interconnection is formally conveyed through citations. Citations testify to the influence and validity of prior research and have conventionally been gauged by their sheer quantity, known as citation counts. Consequently, citations have assumed a pivotal role in academic evaluation, including the ranking of academic institutions [1–6], the allocation of research funding and awards[7–11], and various decision-making processes[12–15] in the scientific community. In recent times, however, a growing body of scholars has posited that not all citations are created equal, challenging the conventional wisdom that treats all citations uniformly [3,16]. This perspective contends that each citation made by a researcher serves a distinct purpose, thereby questioning the blanket utility of citation counts as a measure of research quality. Indeed, numerous studies have confirmed that a significant proportion of references in scholarly works can be characterized as perfunctory or less influential [17,18]. In light of this, an emerging consensus suggests that citations should be evaluated in a nuanced manner, acknowledging their varying degrees of importance [7,8,16]. Several classification schemes have been developed to discern the nature and significance of citations qualitatively [17,18]. These range from content-based [16] and metadata-based approaches [7,19,20] to count-based [21], sentiment-based methodologies [22] and sentiment analysis [23]. Finney [24] was the first researcher who proposed an automatic model to classify citations into seven categories. Garfield [25] summarized 15 reasons for citing papers. The classification of citations into categories such as important and non-important has been pursued vigorously. Zhu et al. [17] performed the pioneer binary classification of citations. This work was enhanced by Valenzuela et al. [16] by utilizing contextual features and categorized the citations into non-important and important categories. Qayyum and Afzal et al. [7] used the Meta-data approach and enhanced the results further. Wang et al. [26] introduced the syntactic and contextual-based approach.

The classification of citations is generally formulated as a machine learning classification problem, where features are extracted from citation contexts and employed in diverse machine learning models. Machine learning, particularly text classification, has gained prominence as a tool for this purpose, with an array of studies striving to enhance its performance. Recent developments have seen the integration of deep learning techniques, including deep neural networks, recurrent neural networks, and convolutional neural networks, into sentiment analysis and citation classification tasks. These deep learning models, when coupled with traditional text representation methods like TF-IDF and word embeddings, have exhibited promising results. Word embeddings, in particular, has become indispensable in representing textual data and is utilized as inputs for machine learning models. These embeddings are, initially popularized by Word2Vec utilizing Neural Networks was published in 2013 [27] and later expanded upon by models like BERT, offer the advantage of encoding semantic meaning into words, enabling mathematical operations on word representations. However, it’s noteworthy that many studies that employ deep learning and word embeddings in citation analysis often emphasize metrics like overall accuracy, F-score, precision, and recall while overlooking the influence of word embedding size. Additionally, these evaluations frequently involve a small size of datasets. This paper seeks to address these gaps by designing and evaluating three deep learning models using the Keras framework and word embedding approaches. Moreover, it explores the impact of different window sizes on citation classification, aiming to determine the optimal window size that maximizes classification model performance. The window size directly influences the amount of context considered when classifying citations. A very small window may not provide enough contexts, while a too-large window may introduce noise. Identifying the optimal size strikes the right balance, ensuring that relevant information is considered. To ensure fair analysis, dataset balance is a crucial consideration, as imbalanced training datasets can mislead machine learning classifiers [28]. As such, this study preprocesses the dataset to achieve balance This paper contributes to the field in the following ways:

- An examination of three deep learning models and machine learning including Convolutional Neural Network (CNN), and Long-Short Term Memory (LSTM) layers,Gated Recurrent Units (GRUs), SVM, Naive Bayes and Decision Tree for the identification of important citations.
- Identifies the optimal window size for classifying in-text citations into important and not important categories. This aspect of our research is crucial as it helps to fine-tune the classification process and improve the overall performance of our models.
- An evaluation of the use of word embedding,specifically Word2Vec and identification of optimal window size for the task of identifying important citations within scholarly articles
- A comparative analysis of deep learning models against common baselines typically used in text classification tasks, shedding light on their efficacy in citation analysis

Overall, our identification of the optimal window size represents a valuable contribution to the field of citation analysis and text classification, offering practical insights that can be leveraged by researchers, institutions, and organizations seeking to improve their citation assessment processes. The rest of the paper is organized as follows. Sect 2 discusses important works related to the current study. Sect 4 presents an overview of the methodology adopted for the current research as well as a detailed description of the dataset and models used for experiments. Results are discussed in Sect 5, while the conclusion and future work are provided in Sects 6 and 7.

## 2 Related work

The analysis of sentiment in scientific paper citations represents an emerging area of research, with a growing body of work addressing the challenge of detecting important citations within scholarly articles. This shift in focus stems from a realization that each citation made by a researcher serves unique purposes, rendering a one-size-fits-all approach ineffective [29]. Garfield [25] was an early pioneer in this field, distinguishing citations by studying researchers’ motivations, ultimately categorizing them into 15 distinct categories. Building upon this, Finney [24] introduced a semi-automatic citation classification method that classified citations into seven types, while Garzone and Mercer [30] took automation further by classifying citations into an impressive 35 different types. Teufel et al. [31] proposed a supervised machine learning approach that divided citations into four categories and 11 subcategories, and Agarwal et al. [32] constructed classifiers employing support vector machines (SVM) and Multinomial Naive Bayes (MNB) to categorize citations. Jurgens et al [33] introduced a machine-learning approach aimed at categorizing citations into seven distinct categories. Hamedani et al. [34] undertook the task of classifying citations into six different classes based on an analysis of the keywords within those citations. Bakhti et al. [35] proposed a novel classification model that combined ontology with convolutional neural networks (CNNs) to categorize citations into six distinctive classes

In recent years, there has been a growing interest in alternative methods of citation classification, particularly in exploring the impact of citations [36]. Bi et al. [37] distinguished between two types of citations, direct citations and indirect citations, to discern the influence of a citation. Zhu et al. [17] presented a model designed to identify citations with significant academic influence on the papers citing them. They introduced the concept of binary citation classification to differentiate between influential and non-influential citations. This classification process involved the use of diverse features, such as in-text count-based, similarity-based, context-based, position-based, and miscellaneous attributes. Their experiments employed a dataset comprising 100 papers from the ACL anthology, which was then transformed into 3,143 paper-reference pairs, with annotations provided by the authors themselves. Notably, their results demonstrated that the in-text citation count feature outperformed other attributes with a Precision score of 0.35. Valenzuela et al. [16] further refined the binary citation classification concept, categorizing citations as either important or non-important, and employed a supervised learning approach to identify the significant citations. Their dataset, drawn from the ACL anthology, consisted of 465 paper-citation pairs, with annotations by the authors. They employed 12 distinct features and utilized SVM and Random Forest classifiers. Their system achieved an impressive F-measure of 0.65, surpassing the performance of the in-text citation count feature, which achieved a Precision score of 0.37 Faiza et al. [7] proposed an innovative technique for classifying citations into categories of importance, particularly useful in cases where the content of papers is not freely accessible. They utilized two annotated datasets and evaluated them using machine learning classifiers, including SVM, KLR, and Random Forest.

In comparison to a previous study that relied on an extensive list of content-based features for classification, Faiza et al. [7] demonstrated superior Precision results of 0.68, utilizing freely available metadata. However, it is important to note that the binary classification results in the state of the art were deemed insufficient for making informed decisions regarding citations. Building upon this work, Aljuaid et al. [22] expanded the concept by incorporating sentiment analysis into the citation classification process, achieving a remarkable precision value of 0.83. Subsequently, Nazir et al. [21] made further contributions to the citation classification domain, increasing precision results to 0.84.

Several researchers have highlighted the potential benefits of considering citation context information to enhance classification performance [38,39]. Additionally, the significance of the similarity between titles and abstracts in assessing the value of citations has been explored [3,7,17]. Zeng and Acuna [40] proposed a bidirectional long short-term memory (Bi-LSTM) network with an attention mechanism and contextual information to identify citation worthiness. Wang et al. [26] leveraged syntactic and contextual information from citations to identify important citations based on two annotated datasets.

Citation sentiment analysis, an emerging field, focuses on evaluating the sentiments expressed by authors toward the papers they cite. An extensive survey by Author [30] delved into the sentiment analysis process, discussing challenges faced by existing methods and presenting an analysis of these methods and their classifications. They concluded that machine learning is the most commonly used method for analyzing citation sentiments in scientific papers. They also noted the limitations of this approach, suggesting that deep learning methods could effectively address the challenge of analyzing sentiment polarity in scientific paper citation[41].

Numerous studies have concentrated on developing robust models to tackle the increasing complexity of big data, applying sentiment analysis to a wide array of applications, from financial forecasting [42] to marketing strategies [43], and even medical analysis [44]. However, there remains a paucity of research dedicated to the evaluation of different deep learning techniques, providing practical evidence of their performance[42,45,46].

Deep learning relies on numerical representations of text; therefore, word embeddings play a pivotal role in capturing the semantic context of citations. Word embeddings facilitating the automatic extraction of linguistic features from citations within research articles, product reviews, service evaluations, and news articles. Automatic feature extraction is essential, as manual feature extraction, particularly from scholarly articles, can be time-consuming due to the complex structure and grammar. Word embeddings have been extensively explored for sentiment analysis as given in Table 1. word embedding enabling the examination and categorization of reviews, opinions, and attitudes in various domains [47].

**Table 1:** List of studies on citations intent analysis using deep learning.

No	Year	Study	Method	Assessment
1	2017	Paredes et al. [48]	CNN + Word2vec	Precision = 88.7%, Fixed window size may not capture contextual variations in text.
2	2017	Ain et al. [49]	CNN, RNN, DNN	Deep learning networks outperform SVMs, but the comparison is limited to one dataset, reducing generalizability
3	2017	Gupta et al. [50]	LSTM-based deep learning	LSTM-based model improves emotion classification, but moderate inter-annotator agreement (0.59) suggests inconsistencies in labeling, which may affect model training. Struggles with context-dependent meanings.
4	2018	Jangid et al. [51]	CNN, LSTM, RNN	F1 score = 0.69, but reliance on pretrained embeddings could introduce biases
5	2018	Zhang et al. [52]	CNN, DNN, RNN, LSTM	Proposes solutions to improve traditional embeddings, but lacks practical benchmarks or exploration of real-world challenges, such as multilingual or domain-specific applications
6	2018	Schmitt et al. [53]	CNN, LSTM	CNN + fastText embeddings produce strong results, but the use of only one dataset (GermEval 2017). Window size not explored.
7	2018	Li et al. [54]	SRN, LSTM, and CNN	Attention mechanisms may improve model performance, but increased complexity is not always beneficial.
8	2018	Sohangir et al.[55]	LSTM, doc2vec, CNN	CNN outperforms other models, but does not explore more recent transformer-based models, which could potentially outperform CNN
9	2019	Yang et al. [56]	Coattention-LSTM, Coattention-MemNet,	Coattention mechanism improves sentiment feature extraction, but struggles with complex word relationships like negation and implicit sentiments.
10	2019	Abid et al. [57]	CNN, RNN	Accuracy = 90.59% across datasets, but heavy reliance on pre-trained embeddings and high computational cost may limit scalability.
11	2019	Do et al. [58]	CNN, LSTM, GRU	Deep learning reduces reliance on feature engineering, but does not address challenges such as computational costs or the need for large labeled datasets.
12	2020	Dang et al.[59]	RNN, CNN, LSTM	CNN is efficient in terms of accuracy and computational cost, but does not explore the window size parameter
13	2020	Cen et al.[60]	RNN, CNN, LSTM	CNN achieves 88.22% accuracy, but the paper lacks critical analysis of accuracy implications and how text is converted to numerical form.
14	2022	Karim et al. [61]	CNN, LSTM, Fasttext	CNN + fastText achieves 93.7% accuracy, but CNN may overfit if not tuned, and window size is not discussed.

Navigating the selection of an optimal word embedding and deep learning strategy for text analysis can pose substantial challenges, primarily stemming from the varying sizes of datasets, window sizes, and ultimate objectives. selection of optimal window size of embedding is a time-consuming and hard process .Utilizing a window size that is too similar may result in the omission of significant contextual information, potentially limiting the effectiveness of the model. Conversely, opting for a larger window size may lead to an increased likelihood of negative sampling, thereby impacting the overall performance and efficiency of the language processing system. Striking the right balance between window size and context is pivotal for achieving optimal results in various NLP tasks, such as sentiment analysis, information retrieval, and machine translation. This research introduces an innovative content-centring approach to address these challenges in the context of binary citation classification, with a specific focus on sentiment analysis within in-text citations.

## 3 Problem formulation

The problem addressed in this paper revolves around selecting the optimal window size for embedding and deep learning models to classify citations into two distinct categories: “Important” and “Not Important.”

The mathematical representation of this problem can be denoted as follows:


R:(C,t,w)→l,w∈{2,3,5,8,10,12,15}

Where:


$$\begin{array}{llll} C & \text{ represents a citation,}\; & & \;\\ t & \text{ signifies the citation intent,}\; & & \;\\ w & \text{ corresponds to the window size.}\; & & \; \end{array}$$

The objective is to construct a deep learning model, denoted as *γ*, that maps the input (*C*, *t*, *w*) to an output *l*. Here, *l* is a binary label taking values from the set {0, 1}. It essentially indicates whether the citation intent *t* within citation *C* is deemed important (i.e., *l* equals 1) or not (i.e., *l* equals 0).where *w* indicate citation window size, by window size we means here the size of the left/right context window, indicating a specific number of words preceding and following a target word.The literature review reveals a lack of clear consensus on the appropriate window size for linguistic analysis. Some researchers employed a fixed window length, while others experimented with various window sizes tailored to their specific tasks. The default window size, often set at 5 [62], was used by some researchers. Caselles et al. [62] investigated multiple window sizes, ranging from 3, 7, 12, 15, highlighting the task and dataset dependence for optimal window selection. Adewumi et al. [63] achieved favorable results with a window size of 8, chosen from options of [4,8]. Conversely, Levy et al. [64] advocated for a window size of 5 to capture broad topics, while smaller sizes like 2 offered more focused information, albeit potentially missing crucial details; a view corroborated by Mikolov et al.[65], who found a window size of 15 outperformed 5. MacAvane et al. [66] found poor performance with a window size of 5 but success with 2. Lin et al. [67] determined that a window size of 1 was optimal among 1, 2, 4, 8, 16. Bansal et al. [68] concluded that a window size of 10 yielded superior results from options 1, 2, 5, 10. [69,70] reported enhanced sentiment analysis results with a window size of 3. Galea et al. [70] emphasized dataset-dependent hyper-parameter choices within 3, 5, 8. Agrawal et al. [71] reported substantial improvements with a window size of 5 chosen from 3, 5. Levy et al. [72] achieved precision enhancement with a window size of 2. Uygun et al. [73] identified a window size of 3 as optimal among 2, 3, and 5. Rengasamy et al. [74] saw improved system performance with a fixed window size of 8.Based on the aforementioned findings, we opted to select the window size that demonstrated the best performance in our experiments. The purpose is to test the models rigorously and identify the optimal window size.

In simpler terms, this approach entails training independent binary classifiers for each citation label. When presented with an unseen sample, each of these binary classifiers predicts whether the underlying citation holds importance or not. The combined model subsequently predicts all labels for this sample for which the respective classifier yields a positive result.

By creating these models for various citation intents *t*, the paper evaluates the performance of the proposed solutions with the ultimate aim of identifying the combination of deep learning layers and word embedding window sizes that can most effectively capture the distinctive characteristics of text to identify important citations.

## 4 Research methodology

**Fig 1:**
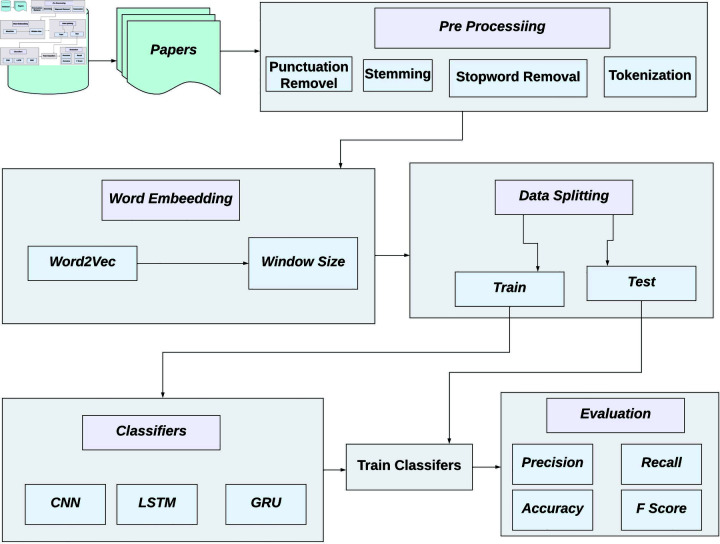
The architecture of the proposed methodology for in-text citation sentiment analysis for identification of important citation.

In this section, the fundamental model of the proposed approach for the identification of important citations is presented. Additionally, the word embedding and citation classification techniques employed to discern citation intent are elucidated. Fig 1 illustrates the architecture of the innovative citation intent labeling technique. To populate the dataset, research articles were gathered and meticulously selected for relevance. From these articles, the citation context was extracted, specifically focusing on in-text citations. Subsequently, the citation sentences were subjected to a comprehensive pre-processing regimen. Word embedding techniques were then harnessed, experimenting with various window sizes, to craft citation context vectors. In the ultimate phase, these embedding vectors were input into the classification techniques for further analysis and assessment.

### 4.1 Proposed algorithm

**Purpose:** The purpose of this algorithm is to find the optimal window size by trying different window sizes using deep and machine learning models for important citation identification. The algorithm selects the window size on which the model achieved the best precision and accuracy.

**Input:**

1. Dataset *D* containing citations.
2. Parameters: *window_sizes, deep_learning_models*.

**Algorithm:**

1. **selected_citations** ← [] // To store selected citations.
2. **for each citation *c* in *D*:**
  i. **if meets_selection_criteria (*c*):**
    (i) **preprocessed_citation** ← preprocess_citation(*c*)
    (ii) **for each** *window*_*size* **in** *window*_*sizes*:
      (A) **numeric_representations** ← convert_to_numeric_representations (preprocessed_citation, window_size)
      (B) **balanced_dataset** ← balance_dataset (numeric_representations)
      (C) **for each** *model_type* **in** *deep_learning_models*:
      (D) **model** ← initialize_model(*model_type*)
      (E) **trained_model** ← train_model(*model, balanced_dataset*)
      (F) **predicted_labels** ← []
      (G) **for each unseen_citation** $C^{\prime }$ **in** *D*_*unseen*:
      (H) **preprocessed_unseen_citation** ← preprocess_citation($C^{\prime }$)
      (I) **numeric_representation** ← convert_to_numeric_representation (Unseen_citation, window_size)
      (J) **prediction** ← predict_label(trained_model, numeric_representation)
      (K) **predicted_labels.append(prediction)**
      (L) **store_results(predicted_labels)**
    (iii) **else:**
      (A) // Handle cases where the citation does not meet the selection criteria
      (B) **continue** // Skip to the next citation
3. **return best_results()** // Return the results with the best precision and accuracy

The dataset is iterated through, each citation is reprocessed, different window sizes are experimented with, the dataset is balanced, various deep learning models are trained, labels for unseen citations are predicted, and the results are stored. Finally, the best combination of model, window size, and pre-processing is returned based on the evaluation criteria.

### 4.2 Dataset description

In this phase of the study, data extraction is carried out using two widely recognized benchmark datasets: ACL-ARC and SciCite, which are extensively employed for citation classification tasks.

#### 4.2.1 SciCite

The first dataset utilized is SciCite, originally developed by Cohen et al. [75]. Several factors influenced our choice of the SciCite dataset:

1. It is a well-established, publicly accessible dataset specifically tailored to the domain of computer science citations
2. The dataset boasts a substantial volume of data, which greatly contributes to robust analysis.
3. SciCite has a well-documented history of use in cutting-edge approaches for citation classification tasks.

However, it’s worth noting that the SciCite dataset presents an imbalanced class distribution. Furthermore, it provides rich details, including the section name housing the in-text citation, the IDs of both citing and cited papers, the citation context, and crucially, the class label for citation intent. The class labels of dataset are “background,” “result,” and “introduction”. However, these labels are further transformed into binary classes, represented as 0 and 1, following the definition of important and not important classification provided by Valenzuela et al. [16].

#### 4.2.2 ACL-ARC.

The second dataset employed is ACL-ARC, assembled by Valenzuela et al. [16]. This dataset encompasses roughly 465 records and offers invaluable insights for analysis. It includes comprehensive information such as citation context, in-text citation locations, paper IDs, publication years, paper titles, author IDs, section titles, section numbers, the phrase preceding the citation context, and, significantly, the citation purpose. Citation purposes are defined through class labels like background, usage, comparison, inspiration, extension, and future work. The dataset is accessible at https://allenai.org/data/data-all.html. Annotators categorized citations into four distinct groups based on their significance. Group 0 represented related work, group 1 represented comparisons, group 2 indicated utilization of the work, and group 3 suggested extensions of the work. These four groups were subsequently amalgamated into two categories. The consolidation of categories 0 and 1 resulted in label 0, denoting non-important work, while the merger of categories 2 and 3 yielded label 1, signifying important citations.the detail of datasete is listing in Table 2.

**Table 2:** Details of intext-citation sentiment corpus.

Attribute	No of Records
SciCite Dataset	
No of in-text Citations	7779
No of important instances	3276
No of Not important instances	4503
ACL-ARC Dataset	
No of in-text Citations	465
No of important instances	398
No of Not important instances	67

### 4.3 Addressing imbalanced data.

The initial dataset exhibited a pronounced class imbalance, with a significantly higher number of instances in the majority class compared to the minority class. This imbalance posed a challenge for model training, as it could lead to biased predictions favoring the majority class, thereby compromising the model’s performance on the minority class. To mitigate this issue, the Synthetic Minority Oversampling Technique (SMOTE) was employed as the primary method for balancing the dataset.

SMOTE is widely regarded as the “de facto” standard for handling imbalanced datasets due to its simplicity and robustness across diverse problem domains. Since its introduction in 2002, SMOTE has demonstrated success in various applications. The algorithm addresses class imbalance by generating synthetic samples for the minority class. Specifically, it identifies the *k*-nearest neighbors for each instance in the minority class and generates new synthetic examples by interpolating between the instance and its neighbors along the connecting line segments.

In this study, *k* = 5 was used to generate synthetic samples, meaning that for each minority instance, five synthetic samples were created. The SMOTE algorithm was implemented using the *imbalanced-learn* library in Python, enabling seamless integration into the data pre-processing pipeline. This approach significantly improved class balance within the training dataset, enhancing the model’s ability to learn effectively from both classes.

The impact of SMOTE on imbalanced datasets has been validated in prior research. For instance, [85] applied SMOTE for toxic comment detection and reported an accuracy improvement of 97%, demonstrating the technique’s efficacy in addressing class imbalance challenges. In a prior study [76], researchers employed sampling techniques like SMOTE-ENN to mitigate the effects of imbalanced data.

### 4.4 Citation context pre-processing

Pre-processing plays a pivotal role in text classification tasks and involves several essential steps to prepare the text for further analysis. Uysal and Gunal [66] extensively discussed four common text classification steps: stop word removal, tokenization, case conversion, and stemming/lemmatization. In our specific context, we focus on citation contexts extracted from research papers within our dataset.

Tokenization is the first step in pre-processing, which involves breaking down text, such as paragraphs and sentences, into individual words or tokens. This is essential for conducting a more detailed analysis of the text, enabling us to understand the linguistic relationships between words. Tokenization provides the foundation for various subsequent tasks in natural language processing (NLP), such as sentiment analysis, which can depend on the arrangement of words in a sentence.

Following tokenization, punctuation removal is performed. This step involves identifying and removing punctuation marks such as periods, commas, semicolons, and parentheses. While punctuation is important for sentence structure and readability, it does not significantly contribute to the meaning when processing text for NLP tasks like citation context analysis.

Next, stop word removal is applied. Stop words are words that appear frequently in text but are typically irrelevant to the main topic, such as prepositions, conjunctions, and articles. Stop word removal is language-specific, and in our case, we used the Natural Language Toolkit (NLTK) [67] in Python, which provides an extensive list of predefined stop words in 16 languages. We also supplemented NLTK’s list by adding certain numbers and special characters that do not affect sentence meaning.

The next step involves case conversion and function word removal. This process converts all uppercase words to lowercase, ensuring uniformity in the dataset, regardless of the words’ positions or forms. This step eliminates inconsistencies that may arise from different casing conventions, such as distinguishing proper nouns from regular words.

Finally, stemming or lemmatization is performed. While stemming reduces words to their root forms (e.g., “running” becomes “run”), lemmatization ensures that the resulting word is a valid dictionary word. Stemming is language-specific, and various algorithms exist to perform it effectively [70]. In our study, we opted for the WordNet Lemmatizer from NLTK, which yielded better results compared to stemming. Stemming or lemmatization significantly impacts the effectiveness of word embedding techniques, as it ensures that different forms of a word are treated as the same word, improving the representation.

After completing these pre-processing steps, we were ready to apply word embedding techniques to generate vectors that represent the cleaned citation contexts.

After completing the pre-processing stage, we were well-prepared to apply the word embedding technique to generate vectors representing the cleaned citation context.

### 4.5 Word embedding

To quantify the relationships between individual words within a citation context and across citation sentences, we transform textual representations into numerical forms. This conversion allows for the effective application of machine learning algorithms to text. Word embedding plays a pivotal role in this process, representing words numerically in a dense format where similar words share similar learned representations. Word embedding constitutes a significant advancement in applying deep learning to natural language processing tasks. The selection of an appropriate word embedding method is crucial as a pre-processing step in natural language processing tasks, such as text classification. Baroni et al. [77] conducted a comparison of frequency-based and prediction-based approaches, validating the claim that prediction-based methods excel in various scenarios. Hence, we exclusively opt for prediction-based word embedding techniques, including Word2Vec.

#### 4.5.1 Word2Vec.

**Fig 2:**
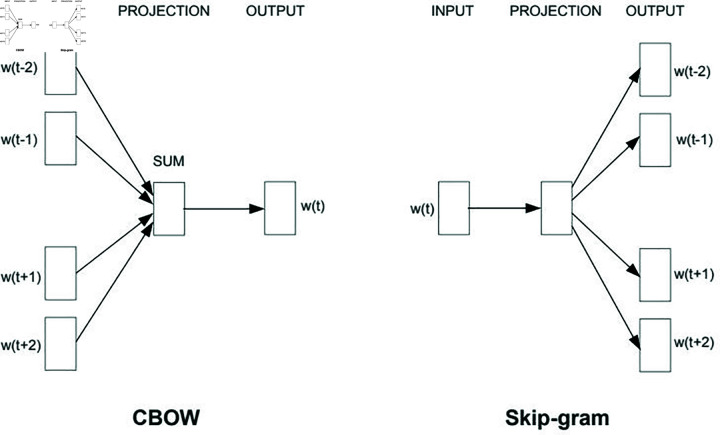
General architecture model (based on conventional frameworks). The CBOW model architecture predicts the current word based on the context, and the Skip-gram model predicts surrounding words given the current word.

Word2Vec is a widely used method for text representation that predicts the likelihood of word distributions based on neighboring words. It comprises two deep learning architectures: the Continuous Bag of Words (CBOW) and the Skip-Gram model. In the CBOW architecture, the probability of neighboring words is predicted based on a center word, whereas the Skip-Gram model predicts the probability of the center word based on its neighboring words. The architecture of the Word2Vec model is illustrated in Fig 2. Eq 1 describes how word probability estimation is performed using neighboring words, and Eq 2 demonstrates the representation process in Word2Vec.


f(x)=1T ∑t=kT−k log ⁡ p(wt∣wt−k,… ⁡ ,wt+k) (1)

$$\begin{array}{c} y=U\cdot h(w_{t-k},\ldots ,w_{t+k};W)+b \end{array}$$ (2)

To prepare the text for processing, it is converted into a numeric representation using Word2Vec with varying window sizes, including 2, 3, 5, 8, 10, 12, and 15.

### 4.6 Modeling methods

Three deep learning models are presented that utilize CNN, GRU, and LSTM, along with three machine learning models, SVM, Decision Tree, and Naive Bayes. All of these models are available within the Keras framework^1^. Each model features the same number of layers to ensure a fair comparison of their performances based solely on the type of neural network they employ.

Machine learning (ML) and deep learning (DL) models differ significantly in their methodologies and performance across varying datasets. ML models typically depend on manual feature engineering and employ relatively simpler algorithms, such as decision trees and support vector machines, with their effectiveness largely influenced by the quality and relevance of the engineered features. Conversely, DL models utilize multi-layered neural networks that automatically learn hierarchical feature representations from raw data, making them particularly well-suited for tasks involving large datasets and complex patterns. The performance of these models varies based on dataset characteristics: ML models often exhibit superior performance on smaller datasets, as DL models require substantial data to prevent overfitting. However, DL models excel on large datasets, as they can capture intricate patterns without relying on extensive feature engineering. Moreover, ML models tend to perform better on structured datasets with clearly defined features, while DL models are more effective in processing unstructured data, such as images and text, due to their ability to model complex and nuanced relationships. For instance, Alqahtani et al. [78] employed ML and DL algorithms, including long short-term memory (LSTM), artificial neural networks (ANN), and gated recurrent units (GRU), for text classification tasks. Their findings indicate that LSTM achieved an accuracy of 92%, outperforming other models and baseline studies. Arshad et al. [79] utilized a deep learning model for the identification of important citations and achieved a precision score of 97% using a Convolutional Neural Network (CNN).

It is important to note that the input and output layers among these models are identical to facilitate a direct performance comparison. More precisely, the input layer is an Embedding layer responsible for mapping the words from the input text to their respective word embeddings. The final layer is a Dense layer that translates the intermediate model outputs into a single label, which can only assume the values 0 and 1.

#### 4.6.1 Convolutional Neural Network (CNN).

Convolutional Neural Networks (CNNs) are powerful deep neural networks known for their effectiveness in handling large-sized data [80]. CNNs efficiently learn complex features through the application of convolution, nonlinear activation, dropout, and pooling layers [81]. In CNNs, training occurs in an end-to-end fashion, which enhances efficiency. To encode semantic information, fully connected layers are employed at the end of the model. CNNs are feed-forward networks in which filters are applied to the output of the preceding layer to map features. The primary components of a CNN model include convolutional layers, pooling (or sub-sampling) layers, a flatten layer, an activation function (often ReLU), a dropout, and a fully connected layer. Convolutional layers extract local and high-level features by assigning weights to kernels during training. Pooling layers reduce overfitting by reducing the dimensionality of features mapped by convolutional layers. Common types of pooling layers are max-pooling and average-pooling, with max-pooling selecting sharp features over average-pooling. In this study, the rectified linear unit (ReLU) activation function is used is given in Eq 3:


$$\begin{array}{c} y=\max(0,i) \end{array}$$ (3)

where y represents the activation output and i represents the input. Convolution layers extract local and high-level features by assigning weights to the kernel during the training phase. For binary classification tasks, the cross-entropy error is often used as the loss function. This has also been used in this study, computed as given in Eq 4.


$$\begin{array}{c} \text{cross-entropy}=-(i\cdot \log(p)+(1-i)\cdot \log(2-p)) \end{array}$$ (4)

where *i* represents the indicator of class labels, a *log* is a natural log, and *p* represents the probability that is predicted. While CNNs were originally designed for image classification, they have found applications in text categorization, including text sentiment analysis [82], text summarization [83], and text report classification [84]. In this study, CNNs are utilized for citation classification.

#### 4.6.2 Long Short-term Memory (LSTM).

**Fig 3:**
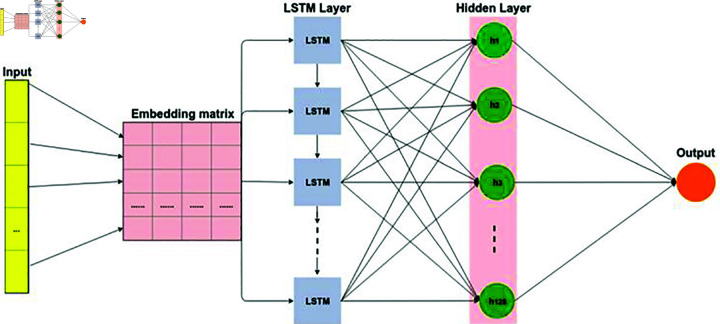
Architecture of LSTM Model used for citation analysis.

Fig 3 illustrates a model that employs the LSTM layers for binary text classification. LSTMs extend the capabilities of Recurrent Neural Networks (RNNs) and are specifically designed to work with sequences. They use memory blocks to capture the state of computation, enabling them to learn temporal dependencies within data sequences [51]. LSTMs are proficient at associating the current data chunks with previously processed data chunks, allowing them to infer semantic patterns describing the history of input data [52]. This addresses a common limitation of standard RNNs, which heavily rely on the most recent input data..

#### 4.6.3 Gated Recurrent Unit (GRU).

It is another variant designed to mitigate the vanishing gradient problem associated with standard RNNs. GRUs exhibit similar design principles to LSTMs and can achieve equally impressive results in various applications [57]. All three models—CNN, LSTM, and GRU—are utilized in this study, and their performance is compared.

### 4.7 Experimental setup

The main goal of this study was to compare the performance of various deep learning and machine learning models across different window sizes for classifying citations as either important or not important. To maintain consistency across experiments, all models were trained using the same pre-processed datasets, which included citation contexts extracted from research papers. The evaluation focused on the models’ ability to accurately classify citation importance.

The pre-processing steps, including tokenization, stop word removal, punctuation elimination, case normalization, and lemmatization, were applied uniformly to all citation contexts to ensure the data was ready for model input. This pre-processing aimed to provide clean and standardized input for both the deep learning and machine learning models.

#### 4.7.1 Hyper-parameter settings.

For consistency across all models, the following hyper-parameter settings were applied:

- **Learning Rate:** A learning rate of 0.001 was used for all models to balance convergence speed and stability.
- **Batch Size:** A batch size of 32 was selected to ensure efficient training while preventing memory overload.
- **Epochs:** Each model was trained for 100 epochs, with early-stopping applied if the validation accuracy did not improve for 5 consecutive epochs.
- **Optimizer:** The Adam optimizer was used for all models due to its robust performance in handling sparse gradients.
- **Dropout Rate:** A dropout rate of 0.5 was employed in CNN, LSTM, and GRU models to mitigate overfitting.

#### 4.7.2 Evaluation metrics.

Model performance was assessed using standard evaluation metrics such as accuracy, precision, recall, and F1 score, which are commonly used in classification tasks. These metrics were calculated based on the confusion matrix, which includes true positives (TP), false positives (FP), true negatives (TN), and false negatives (FN):


$$\begin{array}{ccc} & \text{Accuracy}=\frac{T_{p}+T_{n}}{T_{p}+T_{n}+F_{p}+F_{n}} & \end{array}$$ (5)

$$\begin{array}{ccc} & \text{Precision}=\frac{T_{p}}{T_{p}+F_{p}} & \end{array}$$ (6)

$$\begin{array}{ccc} & \text{Recall}=\frac{T_{p}}{T_{p}+F_{n}} & \end{array}$$ (7)

$$\begin{array}{ccc} & \text{F-Measure}=\frac{2\times (\text{Precision}\times \text{Recall})}{\text{Precision}+\text{Recall}} & \end{array}$$ (8)

Categorizing TP, FP, TN, and FN as the metrics for true positive, false positive, true negative, and false negative, respectively, these values are obtained from the confusion matrix of each classifier. Ultimately, we conducted a performance evaluation across various model-embedding combinations and meticulously examined the outcomes to pinpoint the configuration that demonstrated the highest levels of accuracy, precision, and predictive reliability concerning citation importance.

## 5 Results and discussion

For experimentation, the Kaggle environment was used, utilizing a GPU T4*2 accelerator. To implement the deep learning models, the Keras^2^ and TensorFlow^3^ libraries were leveraged, both renowned for their capabilities in machine learning tasks. The experiments involved the deployment of CNN, LSTM, and GRU models, as well as classical machine learning models such as SVM, Naive Bayes, and Random Forest with varying window sizes, as previously detailed. This comprehensive approach allowed for a thorough analysis of the performance of these algorithms when combined with word embeddings and different window sizes. Across all experiments, key parameters were consistently configured. Specifically, the number of epochs was set to 50, the batch size to 32, and the vector dimension to 300. These settings ensured a rigorous and systematic evaluation of the models’ performance.

To gauge the effectiveness of the models, a set of well-established metrics was relied upon. These metrics encompassed accuracy, precision, recall, and the F1-score. Additionally, since the F1-score derives from precision and recall, these two measures were included for reference purposes. Collectively, these metrics provide a comprehensive overview of the models’ performance.

The initial focus was on the Sci-Cite dataset, which contains a substantially larger volume of in-text citations compared to other datasets. To systematically assess the performance of the models, a detailed analysis was conducted. Specifically, different subsets of this dataset were created, each comprising varying percentages of the initial data, ranging from 10% to 100%.

As depicted in Table 3, the experiments encompassed a wide array of combinations involving different window sizes for word embedding and various deep learning techniques. Through this rigorous evaluation, the study aimed to answer critical questions: Which window size for embedding and deep learning models demonstrates optimal performance in the task of identifying important citations? Are the models useful, and how well do they generalize to other datasets?

**Table 3:** Experimental settings combining different embedding, window size and classification.

Setup	Embedding	Window Size	Classification Algorithm
1	Word2vec	{2,3,5,8,10,12,15}	CNN
2	Word2vec	{2,3,5,8,10,12,15}	LSTM
3	Word2vec	{2,3,5,8,10,12,15}	GRU

### 5.1 Comparison of predictive performance of models using dataset-1

Extensive experiments have been carried out for citation sentiment analysis. Efforts are underway to develop an efficient method for in-text citations’ sentiment analysis. Machine learning models and deep learning models used in the experiments are CNN, LSTM, GRU, SVM, Decision Tree, and Naive Bayes. Word embedding techniques, namely Word2Vec and their various combinations, are investigated for citation sentiment analysis.

An exhaustive series of experiments was conducted to delve into the realm of citation sentiment analysis. The overarching objective was to craft an efficient methodology for dissecting the sentiment within in-text citations. To achieve this, a diverse array of machine learning and deep learning models, including CNN, LSTM, GRU, SVM, Decision Tree, and Naive Bayes, was harnessed.

Additionally, the investigation delved into word embedding techniques, specifically focusing on the renowned Word2Vec and FastText approaches. The key focus lay in uncovering the optimal combinations of these techniques for enhancing citation sentiment analysis.

These experiments represent a robust exploration into the domain of sentiment analysis within citations, and their outcomes are instrumental in advancing the understanding of effective methodologies in this critical area.

### 5.2 Evaluation of word embedding models with different window sizes

Initially, the models underwent training using Word2Vec word embedding with various window sizes, as summarized in Table 3. A consistent trend emerged across all machine learning models, highlighting the beneficial impact of enlarging the window size on the obtained results. However, this effect diminishes when the window size becomes smaller than the length of the sentences.

**Fig 4:**
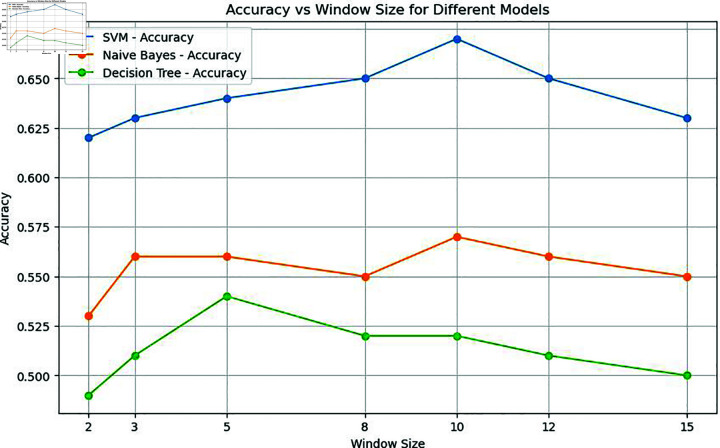
Accuracy of machine learning models with word embeddings across different window sizes, highlighting performance trends.

**Fig 5:**
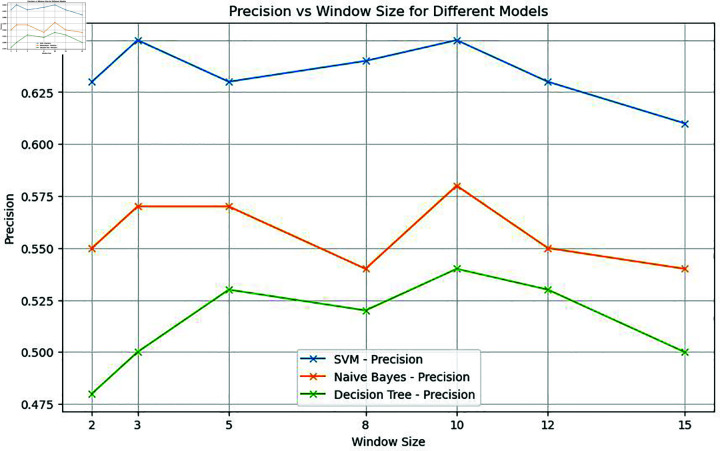
Precision of machine learning models with word embeddings across different window sizes, highlighting performance variations.

**Fig 6:**
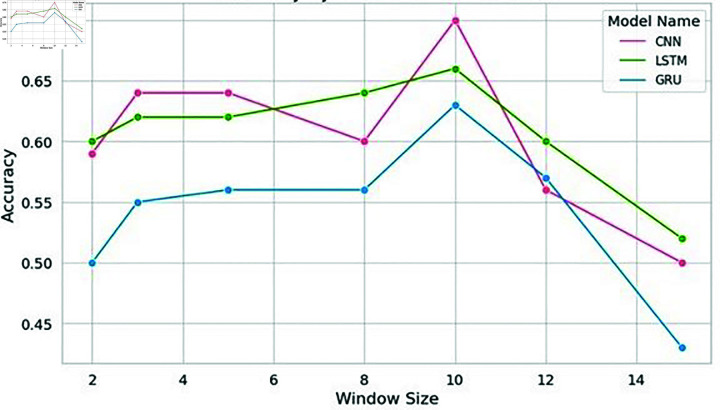
Accuracy of deep learning models with word embeddings across different window sizes, highlighting performance trends.

**Fig 7:**
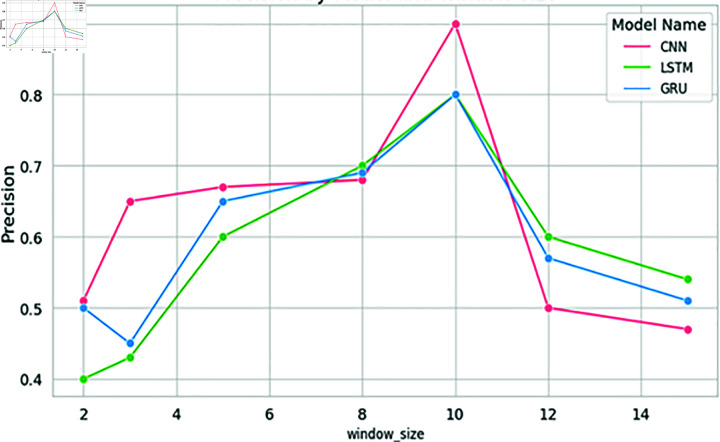
Precision of deep learning models with word embeddings across different window sizes, highlighting performance variations.

In this specific study, the model exhibited optimal performance with a window size of 10. Notably, the classical machine learning model, Support Vector Machine (SVM), displayed a remarkable spike in accuracy at this window size. Nevertheless, as the window size exceeded this threshold, the model’s performance started to decline, as shown in Figs 4, 5, 6, and 7. Similarly, the decision tree algorithm achieved commendable accuracy at a window size of 10. Both the SVM and decision tree algorithms also attained high precision values at this window size, as shown in Fig 5.

These empirical findings were substantiated by the results of the deep learning models, further solidifying the hypothesis that an increased window size positively influences results, as long as it corresponds to or surpasses the sentence length. Strikingly, a window size of 10 emerged as the most favorable choice for identifying significant citations, as shown in Figs 10 and 11. The CNN models showcased impressive accuracy with a window size of 10, while the GRU and LSTM models’ accuracy began to decrease after this point, though they excelled in precision at this setting.

This consensus regarding the favorable window size of 10 remained consistent across both machine learning and deep learning models, reinforcing the robustness of this window size selection and its effectiveness in this research context.

### 5.3 Predictive performance of models using dataset-2

Expanding and enhancing the scope of the research, the analysis was extended beyond Dataset-1 to incorporate the “ACL-ARC” dataset. This expansion allows for rigorous validation of the findings obtained from the classification algorithms when applied to the domain of in-text citation sentiment analysis, with a specific focus on the identification of important citations.

A series of extensive experiments were conducted, leveraging all the features that were instrumental in Dataset-1. The results, detailed comprehensively in Table 12 and onwards, once again underscore the exceptional performance of the proposed model. As demonstrated in the previous dataset, a window size of 10 emerges as the optimal choice, thereby reinforcing the robustness of the approach across different datasets.

### 5.4 Evaluation of word embedding models with different window sizes

In a manner consistent with the approach for Dataset 1, model training was conducted using varying window sizes, specifically {2, 3, 4, 8, 10, and 15}, for Dataset 2. Both traditional machine learning models, such as Support Vector Machine (SVM), Naive Bayes, and Decision Tree, as well as deep learning models, including Convolutional Neural Network (CNN), Long Short-Term Memory (LSTM), and Gated Recurrent Unit (GRU), were employed to classify in-text citations into two classes: important and not important. This classification was based on the evaluation metrics of precision, and accuracy.

**Fig 8:**
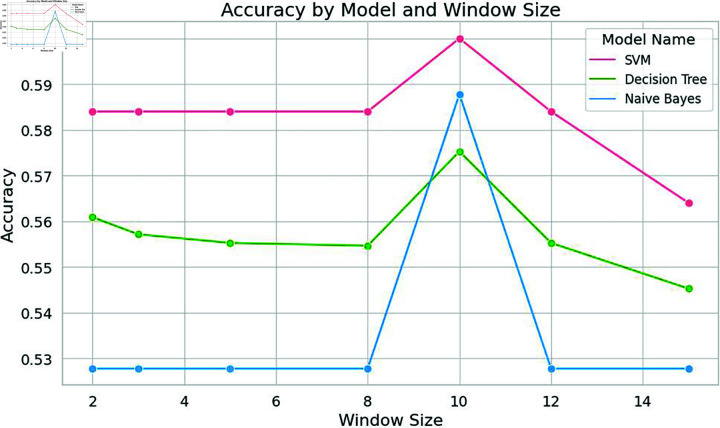
Accuracy of machine learning models with word embeddings across different window sizes using Dataset2, highlighting performance trends.

**Fig 9:**
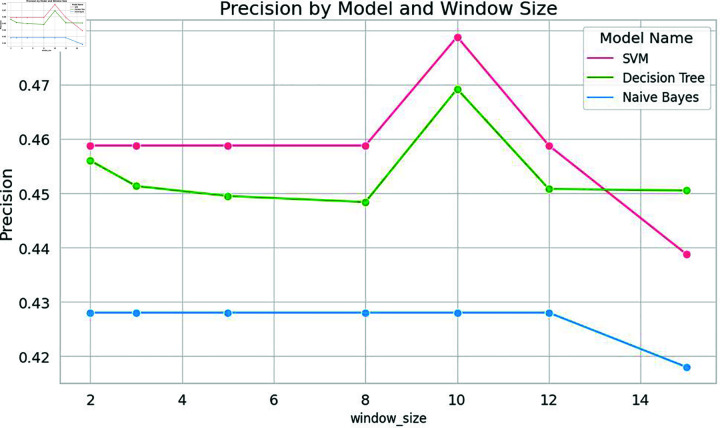
Precision of machine learning models with word embeddings across varying window sizes, highlighting performance patterns.

**Fig 10:**
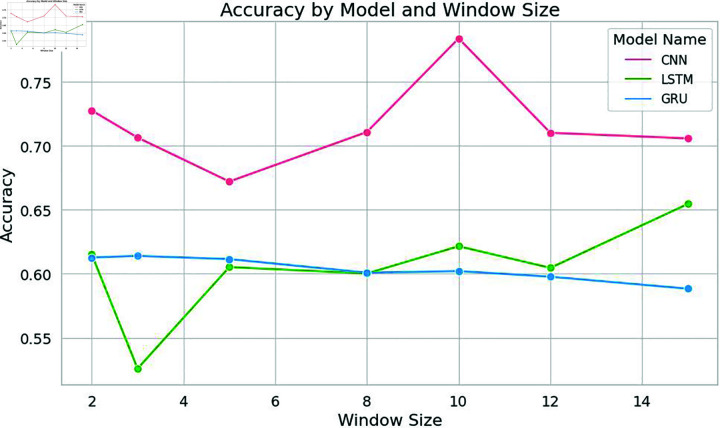
Accuracy of deep learning models with word embeddings across different window sizes using Dataset2, highlighting performance trends.

**Fig 11:**
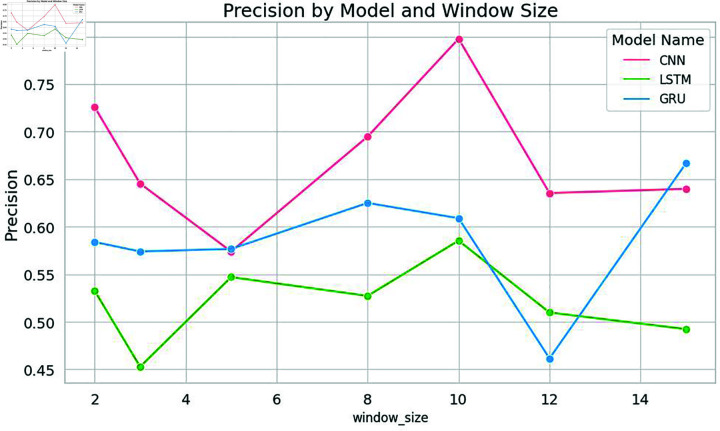
Precision of deep learning models with word embeddings across varying window sizes, highlighting performance patterns.

The results mirrored those obtained for Dataset 1, with Support Vector Machine (SVM) once again demonstrating exceptional performance at a window size of 10. At this window size, SVM achieved notably high precision and accuracy, as shown in Figs 8 and 9. Similarly, Naive Bayes and Decision Tree exhibited high accuracy at a window size of 10, as shown in Fig 8. All models achieved high levels of accuracy and precision at a window size of 10. Among the deep learning models, CNN achieved remarkable accuracy when employing a window size of 10, as shown in Fig 10. The accuracy of the models started decreasing after a window size of 10. Both the LSTM and CNN achieved high levels of precision at a window size of 10, as given in Fig 11.

The results indicate that a window size of 10 is the optimal choice for classification when employing embeddings, as illustrated in Tables 4 and 5. However, it is essential to emphasize that this window size must remain smaller than the length of the sentence to prevent any adverse effects on model performance.

**Table 4:** Performance metrics of deep learning models on different window sizes.

Model	Window Size	Accuracy	Precision
CNN	2	0.59	0.51
	3	0.64	0.65
	5	0.64	0.67
	8	0.60	0.68
	10	0.70	0.90
	12	0.56	0.50
	15	0.50	0.47
LSTM	2	0.60	0.40
	3	0.62	0.43
	5	0.62	0.60
	8	0.64	0.70
	10	0.66	0.80
	12	0.60	0.60
	15	0.52	0.54
GRU	2	0.50	0.50
	3	0.55	0.45
	5	0.56	0.65
	8	0.56	0.69
	10	0.63	0.80
	12	0.57	0.57
	15	0.43	0.51

**Table 5:** Performance metrics of machine learning models across different window sizes.

Model	Window Size	Accuracy	Precision
SVM	2	0.62	0.63
	3	0.63	0.65
	5	0.64	0.63
	8	0.65	0.64
	10	0.67	0.65
	12	0.65	0.63
	15	0.63	0.61
Naive Bayes	2	0.53	0.55
	3	0.56	0.57
	5	0.56	0.57
	8	0.55	0.54
	10	0.57	0.58
	12	0.56	0.55
	15	0.55	0.54
Decision Tree	2	0.49	0.48
	3	0.51	0.50
	5	0.54	0.53
	8	0.52	0.52
	10	0.52	0.54
	12	0.51	0.53
	15	0.50	0.50

### 5.5 Discussion

In this section, outcomes and insights derived from the exploration of state-of-the-art machine learning and deep learning models, combined with word embedding techniques employing varying window sizes, for the purpose of classifying citations into “important” and “not important” categories are presented. The evaluation of word embedding techniques at each window size was conducted using standard performance measures.

It is evident from Fig 4 that when it comes to accuracy, classical machine learning models utilizing word embeddings with varying window sizes, SVM in particular, excel when the window size is set to 10, outperforming other models. Conversely, the accuracy of SVM, Decision Tree, and Naive Bayes models tends to decrease after a window size of 10, as depicted in Figs 4 and 8.

In a similar vein, other machine learning models and the voting classifier demonstrated moderate performance results when trained with embedding at a window size of 10. SVM also exhibited the highest precision and accuracy when the embedding window size was set to 10. Notably, both precision and accuracy for all machine learning models decreased as the window size exceeded 10, as shown in Figs 8 and 9.

In the context of deep learning models, CNN achieved high accuracy when trained with an embedding window size of 10. However, the accuracy decreased as the window size exceeded 10. Furthermore, the precision of all the deep learning models increased notably when trained with an embedding window size of 10 and decreased after window size 10 as shown in Fig 11. Similarly, both the LSTM and CNN models exhibited the highest precision at a window size of 10 as shown in Fig 11.

This consistency in findings across both machine learning and deep learning models underscores the significance of a window size of 10 as the optimal choice for classifying citations.

To validate the findings of the first dataset, the same classification models with embedding techniques were applied to the second dataset D2. The results showed that the accuracy of all machine learning models improved as the window size increased to 10 but declined beyond this point, as illustrated in Fig 8. Similar to the results from the first dataset, SVM exhibited the highest precision at a window size of 10, as seen in Figs 5 and 9. Both Naive Bayes and Decision Tree also achieved their highest precision at a window size of 10. In the case of deep learning models, CNN, GRU and LSTM models achieved their highest precision at a window size of 10 and saw a decrease in precision when the window size exceeded 10, as shown in Fig 11.

In summary, the discussion emphasizes that, in the case of classical machine learning models, SVM performs exceptionally well with an embedding window size of 10, excelling in terms of accuracy and precision for the classification of citations into “important” and “not important” categories. Meanwhile, for deep learning models, CNN, LSTM, and GRU models achieve the best performance with a window size of 10 for this classification task. It is worth noting that the dataset used for model training was initially imbalanced and was subsequently balanced using SMOTE techniques. Additionally, the optimal embedding size was explored for effectively classifying citations into “important” and “not important” categories.

## 6 Conclusion

This research highlights the crucial role of window size in optimizing word embeddings for citation classification, identifying a window size of 10 as the optimal choice for balancing semantic context and noise reduction. Through systematic evaluation, the study demonstrated that this window size consistently delivers the best performance, enabling models like CNN and SVM to achieve the highest accuracy and precision. Smaller window sizes lacked sufficient context, reducing model effectiveness, while larger ones introduced excessive noise, compromising reliability. These findings emphasize the importance of tailoring embedding techniques to the specific needs of citation analysis and optimizing input features for both traditional machine learning and deep learning models. By advancing the understanding of how context length impacts model performance, this research offers critical insights into parameter selection and establishes a strong foundation for refining citation analysis methodologies in future studies.

## 7 Limitations and future research directions

This study has made significant progress in sentiment analysis and citation importance identification, but acknowledges several limitations and opportunities for future research. Ensemble models combining different neural network architectures could improve performance, while alternative embeddings like FastText and GloVe may enhance text representation by capturing diverse linguistic features. Expanding the focus beyond in-text citations to include entire research papers and metadata could provide valuable context, and incorporating multi-modal data, such as visual and structural elements, may yield richer insights. Furthermore, addressing the imbalance in dataset through advanced sampling techniques, class weighting, or specialized loss functions could improve model generalization and reliability. Addressing these areas can significantly enhance the accuracy and robustness of models in this field.

## Footnotes

1 https://keras.io/. Accessed on 11th February 2021.

2 https://keras.io.

3 https://www.tensorflow.org/.
